# Delirium: Prevention, Diagnosis, and Management in an Organic Mental Health Ward

**DOI:** 10.1192/bjo.2025.10602

**Published:** 2025-06-20

**Authors:** Haris Hussain, Cara Webb

**Affiliations:** Mersey Care NHS Trust, Warrington, United Kingdom

## Abstract

**Aims:** Delirium is a serious condition affecting hospitalised and long-term care patients. Adherence to NICE guidelines is essential for timely identification and management. This audit aimed to evaluate compliance with NICE guidelines on delirium prevention, diagnosis, and management within a secondary care setting. Specific objectives included assessing whether new patients were screened for delirium risk factors, ensuring that at-risk patients were reviewed within 24 hours, confirming the use of the ‘4AT’ assessment tool where required, and verifying the provision of patient information leaflets.

**Methods:** A retrospective audit was conducted on all inpatients at Kingsley Ward from 21/10/24 to 01/11/24. A total of 18 patients were included in the sample. Data were extracted from the in-house assessment database and electronic system. Key compliance measures included documentation of delirium risk assessments within 24 hours of admission, regular reviews in morning clinical meetings, appropriate use of the ‘4AT’ tool, and provision of patient information leaflets. Compliance was assessed based on recorded documentation in the electronic records.

**Results:** Overall compliance with NICE guidelines for delirium management was 89.1%, demonstrating significant assurance. Key areas of adherence included timely screening at admission and appropriate documentation of delirium assessments. Majority of the criteria achieved a 100% compliance score. However, inconsistencies were identified in clinical documentation, particularly regarding the recording of delirium status. Compliance with Criterion 7.3: “During daily clinical review meetings each patient will be discussed by the MDT to review whether they have developed any signs of delirium”, was 88.1%, slightly below the expected standard. Challenges included multiple authors entering data inconsistently in meeting records, leading to variations in documentation quality.

**Conclusion:** The audit demonstrated high compliance with delirium management guidelines, but areas for improvement remain. To enhance documentation accuracy, a single designated person should complete the log of clinical morning meetings daily, or a verbal handover should be provided when multiple authors are involved. Increased staff awareness and training on the importance of accurate delirium documentation is recommended. Additionally, a dedicated delirium section should be incorporated into the meeting documentation proforma to standardise reporting. By addressing these areas, further improvements in adherence to NICE guidelines and overall patient care quality can be achieved.

